# Liquid Biopsy Detects Relapse Five Months Earlier than Regular Clinical Follow-Up and Guides Targeted Treatment in Breast Cancer

**DOI:** 10.1155/2019/6545298

**Published:** 2019-09-10

**Authors:** Fiona Tsui-Fen Cheng, Nina Lapke, Chin-Chu Wu, Yen-Jung Lu, Shu-Jen Chen, Pei-Ning Yu, Yen-Ting Liu, Kien Thiam Tan

**Affiliations:** ^1^Breast Cancer Center, Shin Kong Wu Ho-Su Memorial Hospital, Taipei City, Taiwan; ^2^ACT Genomics Co., Ltd, Taipei City, Taiwan; ^3^Department of Diagnostic Imaging, Shin Kong Wu Ho-Su Memorial Hospital, Taipei City, Taiwan

## Abstract

Genetic alterations in circulating tumor DNA (ctDNA) are an emerging biomarker for the early detection of relapse and have the potential to guide targeted treatment. ctDNA analysis is often performed by droplet digital PCR; however, next-generation sequencing (NGS) allows multigene testing without having to access a tumor sample to identify target alterations. Here, we report the case of a stage III hormone receptor-positive breast cancer patient who remained symptomless after receiving surgery and adjuvant chemotherapy. Liquid biopsy analysis by NGS revealed the presence of a ctDNA *PIK3CA* N345K mutation five months before the detection of relapse with multiple liver metastases by regular clinical follow-up. To date, clinical implications of the *PIK3CA* N345K variant remain insufficiently investigated; however, everolimus treatment resulted in the shrinkage of tumor lesions and decreased the levels of tumor markers. Four months after treatment initiation, a second ctDNA analysis suggested a relapse, and the patient clinically progressed after five months of everolimus therapy. This case report demonstrates the value of ctDNA analysis by NGS for the early detection of relapse in breast cancer patients. The study further indicates its usefulness for the choice of targeted treatments, suggesting that the variant *PIK3CA* N345K might be associated with everolimus sensitivity.

## 1. Introduction

Liquid biopsies are an emerging biomarker for the early detection of relapse in cancer patients. In breast cancer patients, some studies have obtained encouraging results, although further studies are needed to fully evaluate the potential of liquid biopsies in clinical practice. Two studies with small to intermediate cohort size demonstrated that genetic alterations enable physicians to detect relapse months before the detection by other follow-up methods [1, 2]. In these studies, target mutations were first identified by sequencing of tumor samples, followed by ctDNA detection by droplet digital PCR (ddPCR). The advantage of using next-generation sequencing (NGS) rather than ddPCR is that no tumor sample is needed to identify target alterations and multigene panels can cover a high proportion of patients. Until now, studies using NGS for follow-up in breast cancer patients remain limited.

Another application of liquid biopsies is the identification of mutations that may be associated with treatment outcome. Everolimus is a targeted agent approved for hormone receptor-positive (HR+), HER2− breast cancer patients. It inhibits signaling through the mTOR pathway. However, biomarkers of everolimus response are not well established. A candidate biomarker is *PIK3CA* mutation. Whereas no pronounced difference in the outcome for the addition of everolimus to exemestane was observed between HR+ HER2− patients with or without *PIK3CA* mutations [3], its addition to trastuzumab and chemotherapy has been shown to be more effective in HER2+ patients who harbored *PIK3CA* mutations [4]. These results indicate a different impact of *PIK3CA* mutations depending on breast cancer subtypes, treatment regimens, or both. In addition to evaluating the clinical relevance of the presence of any *PIK3CA* mutation, it is also of interest to analyze potential differences between *PIK3CA* mutation subtypes. The finding that patients with exon 9, but not exon 20, mutations had a particularly good antiproliferative response when everolimus was added to letrozole treatment indicates that mutation subtypes might respond differently to therapy [5].

In this study, we present a case report for which a liquid biopsy sample was obtained months before the regular clinical follow-up. The presence of a *PIK3CA* N345K mutation detected by NGS indicated potential benefit from mTOR pathway targeting agents and enabled the investigation of a potential influence of this mutation subtype on the effectiveness of everolimus therapy.

## 2. Case Presentation

**Figure 1:**
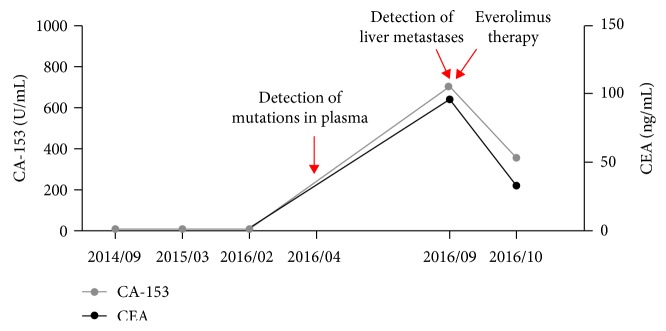
ctDNA analysis detects disease recurrence in a breast cancer patient five months before clinical recurrence. Levels of the tumor markers CA-153 and carcinoembryonic antigen (CEA) are displayed. The detection of ctDNA mutations and liver metastases, as well as the initiation of everolimus therapy, is indicated by red arrows.

**Figure 2:**
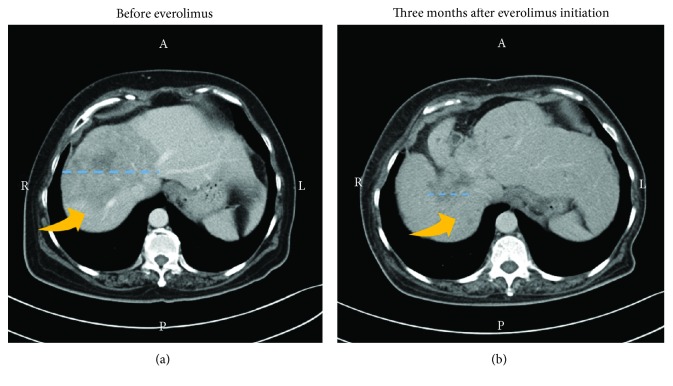
Shrinkage of liver metastasis upon everolimus therapy. A liver CT image is shown for September 2016 displaying a liver lesion for which the location is indicated by a yellow arrow and the diameter marked by a blue dotted line (a). Since the detection of the *PIK3CA* N345K variant indicated an activation of the mTOR pathway, everolimus therapy was initiated. Marked shrinkage of liver metastases was observed by a follow-up CT scan three months after targeted treatment initiation (b).

In March 2014, a 58-year-old woman presented with T2N3M0 stage IIIC estrogen receptor-positive (ER+), progesterone receptor-positive (PR+), and human epidermal growth factor receptor 2-negative (HER2−) breast cancer. She was treated with surgery and adjuvant chemotherapy. For chemotherapy, six cycles of taxotere-epirubicin-cyclophosphamide (TEC) were used. At a clinical follow-up performed one year after initial diagnosis, the patient was without disease symptoms, and there was no evidence of liver metastases by liver echo. After an additional eleven months, a plasma sample was subjected to cell-free DNA (cfDNA) analysis to detect circulating tumor DNA (ctDNA). For analysis, a 12-gene next-generation sequencing panel (ACTMonitor, ACT Genomics) was used. This test performs ultradeep NGS of cfDNA and detects single nucleotide variants and small insertions and deletions in the genes *AKT1*, *BRAF*, *CDKN2A*, *CTNNB1*, *EGFR*, *HRAS*, *IDH1*, *IDH2*, *KRAS*, *NRAS*, *PIK3CA*, and *TP53*. Three genetic variants were detected, namely, *PIK3CA* N345K, *KRAS* Y64H, and the deletion variant *TP53* P177_C182del. Variant frequencies were 1.7%, 0.6%, and 0.2%, respectively. This result indicated the potential of an upcoming relapse. At a follow-up five months later in September 2016, a CT scan indeed revealed multiple liver metastases, and tumor markers were found to be elevated in the blood (Figure 1). Since the detection of the *PIK3CA* mutation indicated an activation of the mTOR pathway, the patient was treated with an oral dose of 10 mg everolimus daily. Upon everolimus therapy, there was a decrease of tumor markers CEA and CA-153 in the blood (Figure 1). Liver metastasis showed remarkable shrinkage after three months of treatment (Figure 2). The patient's disease was under control until January 2017. At that time, a second plasma sample was analyzed. Since an available 8-gene NGS panel was sufficient for tracking the patient's most prominent identified tumor variant, the second sample was analyzed by this test. The used panel includes the genes *CCND1*, *CDH1*, *ERBB2*, *ESR1*, *FGFR1*, *GATA3*, *PIK3CA*, and *TP53*, and therefore allowed the detection of the *PIK3CA* and *TP53* variants, but not the *KRAS* variant. The results showed an increase in mutant allele frequencies, namely, 32.2% for *PIK3CA* N345K and 5.5% for *TP53* P177_C182del, and no new mutations were identified. In February 2017, the disease progressed, as indicated by jaundice and elevated blood tumor markers. Everolimus treatment was stopped, and the patient received eribulin therapy instead.

## 3. Discussion

This report demonstrates that the detection of ctDNA genetic alterations in liquid biopsies can indicate a disease relapse in breast cancer patients long before its diagnosis by regular clinical follow-up. In this case, the lead-time was five months. The case report further indicates that detected genetic alterations may guide the choice of appropriate targeted therapy.

Studies that use NGS as an indicator for disease relapse are limited. However, an extended analysis in one study indicated that it is indeed a feasible method for variant detection [2]. Another study using NGS and enrolling triple-negative breast cancer patients identified ctDNA mutations in four patients, all of whom subsequently relapsed [6]. The average lead-time in these patients was about four months [6]. The present case detected relapse five months before a regular follow-up and represents a further confirmation of the value of liquid biopsies and NGS for early detection of relapse in breast cancer patients.

Due to the approval of mTOR inhibitors for breast cancer, there is a particular interest in *PIK3CA* variants. However, clinical data are limited, especially for variants other than those in exons 9 and 20. The variant *PIK3CA* N345K can be detected in a considerable proportion of breast cancer patients and occurred in about 2.5% of patients from the TCGA cohort [7]. Although the variant is known to be oncogenic [8], its clinical implications remain insufficiently characterized. This case report suggests that patients with *PIK3CA* N345K mutations may benefit from everolimus therapy, as indicated by the shrinkage of liver metastases and decreased blood tumor markers upon everolimus therapy. However, studies with larger patient cohorts are required to confirm this finding.

Our patient finally relapsed five months after treatment initiation. The resistance mechanism leading to relapse remains unclear. It would be interesting to know whether the subclonal variant *KRAS* Y64H detected in the first plasma sample expanded upon therapy, considering that *KRAS* variants have been proposed to lead to everolimus resistance [9]. However, it should be noted that the biological relevance of *KRAS* Y64H is currently unknown.

In conclusion, our case report shows that the analysis of cfDNA by NGS could detect disease relapse in a breast cancer patient five months earlier than routine follow-up. Furthermore, the patient was found to harbor a *PIK3CA* N345K mutation, and the shrinkage of liver metastases upon initiation of everolimus indicated that patients with this genetic alteration might benefit from everolimus therapy.
